# Primary Intracranial Leiomyosarcoma: Report of a Case and Review of the Literature

**DOI:** 10.1155/SRCM/2006/52140

**Published:** 2006-12-24

**Authors:** Sakeer Hussain, Anil Nanda, Marjorie Fowler, Federico L. Ampil, Gary V. Burton

**Affiliations:** ^1^Department of Medicine, Feist Weiller Cancer Center, Louisiana State University Health Science Center - Shreveport, 1501 Kings Highway, Shreveport, LA 71130, USA; ^2^Department of Neurosurgery, Feist Weiller Cancer Center, Louisiana State University Health Science Center - Shreveport, 1501 Kings Highway, Shreveport, LA 71130, USA; ^3^Department of Pathology, Feist Weiller Cancer Center, Louisiana State University Health Science Center - Shreveport, 1501 Kings Highway, Shreveport, LA 71130, USA; ^4^Department of Radiology, Feist Weiller Cancer Center, Louisiana State University Health Science Center - Shreveport, 1501 Kings Highway, Shreveport, LA 71130, USA

## Abstract

A 26-year old man presented with a 3-month history of a progressively enlarging palpable parieto-occipital mass. A CT scan indicated the lesion arose from the dura with bony destruction. A stealth assisted craniotomy was performed with the provisional diagnosis of osteoblastic meningioma. Further histopathologic analysis of the intracranial mass was consistent with leiomyosarcoma. Staging evaluation, including CT and PET scans, demonstrated no other sites of disease. Despite complete surgical resection and radiotherapy to the resection site, the disease recurred locally and systematically 5 months later. Primary intracranial mesenchymal tumors are rare and few cases have been previously reported. Outcomes have been universally poor and current therapeutic approaches appear to have only limited benefit.

## INTRODUCTION

Intracranial neoplasms of mesenchymal origin are uncommon. The majority of these tumors represent metastatic disease from other primary sites. Primary intracranial soft tissue sarcomas can, however, arise from mesenchymal cells of the dura matter or the cerebral blood vessels. These tumors often mimic meningioma on preoperative MRI and, although rare, should be included in the differential diagnosis of dural-based lesions. Immunohistochemical stains can help distinguishing these tumors from meningiomas. Postoperative radiation and chemotherapy should be considered, however, the prognosis has been universally poor. We describe the course of a patient with a primary intracranial leiomyosarcoma and review the literature.

## CASE HISTORY

**Figure 1:**
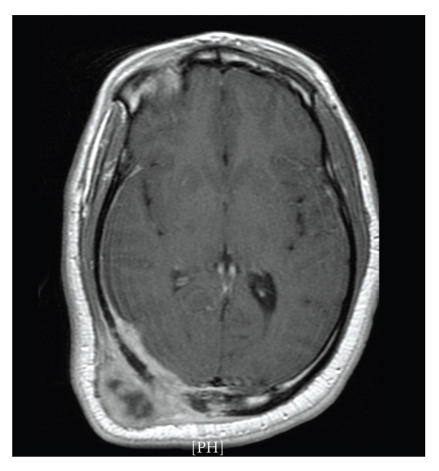
CT scan of the head shows a large vascular soft tissue mass involving the meninges and invasion of right parietal bone.

**Figure 2:**
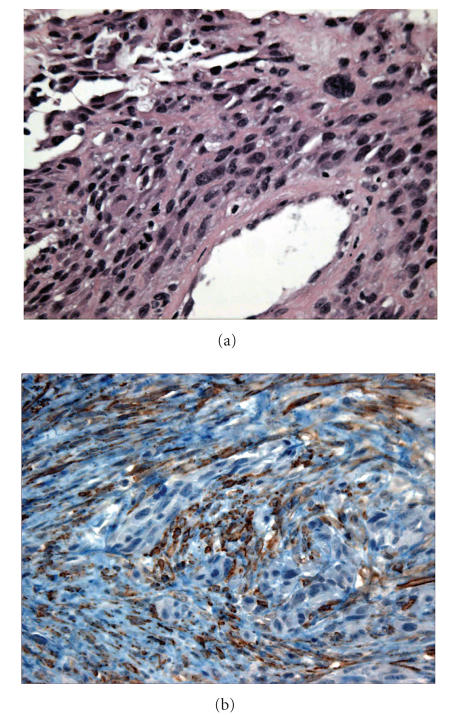
(a) Malignant spindle cells, pleomorphism, and high nucleocytoplasmic ratio. (b) Immunostain positive for smooth muscle actin.

A 26-year old previously healthy male presented with a 3 month history of progressively enlarging parieto-occipital mass. There were no constitutional or neurological symptoms and no symptoms suggestive of other sites of involvement. There was no history of smoking, IV drug abuse, or sexual promiscuity. Physical examination was remarkable for a palpable 5 cm, fixed, nontender mass over the right parieto-occipital region. Complete blood counts and metabolic panel were normal. Viral serology was negative for both human immune deficiency virus (HIV) and Epstein-Barr virus (EBV). A CT scan of the brain demonstrated a large highly vascular soft tissue mass involving the meninges with invasion of the parietal bone and displacement of the brain parenchyma (Figure 1). A clinical diagnosis of osteoblastic meningioma was made. The patient underwent an uncomplicated stealth assisted craniotomy with cranioplasty and gross microsurgical resection of the tumor. The tumor involved the dura mater and had eroded through the bony skull. The surgical margins were negative. The histologic examination demonstrated a malignant spindle cell neoplasm with immunostains positive for smooth muscle actin and negative for epithelial membrane antigen (Figure 2). Pathologic interpretation was a malignant spindle cell neoplasm consistent with high grade leiomyosarcoma with myxoid and epitheloid areas. Staging CT scan of the chest, abdomen and pelvis and PET scan were negative for other sites of involvement. The patient received radiation therapy consisting of 61.8 Gy in 34 fractions using involved field (tumor bed) megavoltage irradiation. Adjuvant chemotherapy was declined by the patient. Five months following the surgery the patient developed pain in the right hip. An MRI of the right hip showed heterogeneous marrow replacement in the right ischium extending to the acetabular marrow, with extraosseous soft tissue component and small ipsilateral joint effusion. A CT scan showed multiple lung lesions and a 2.5 cm liver lesion. An MRI of the brain revealed a suspicious small residual area at the parieto-occipital extradural space. Fine needle aspiration of the lung mass was performed and cytology was consistent with leiomyosarcoma. The patient initially declined systemic chemotherapy, but subsequently received liposomal doxorubicin without response. He died 7 months after the initial diagnosis.

## DISCUSSION

Soft tissue sarcomas are rare tumors and account for only one percent of all cancers [1]. Most intracranial soft tissue sarcomas represent metastatic disease. Primary intracranial sarcomas are extremely rare [2]. Intracranial sarcomas appear to originate from leptomeningeal lining and usually have dural attachment [3]. Pleuripotent mesenchymal stem cells in the dura are probably the cells of origin. Intracerebral sarcomas may also arise from cerebral blood vessel epithelium [4]. These tumors may also originate in the blood vessels outside the dural surface and extend to the skull and meninges. There was no definite evidence to confirm the origin of tumor in this case but involvement of the dura with invasion of the skull suggests a dural origin.

An increased incidence of leiomyoma and leiomyosarcoma has been observed in immunocompromised patients. The association of these neoplasms with Epstein-Barr virus infection and AIDS is well documented in the literature [5, 6]. Our patient, however, had negative serology for both HIV and EBV infections. Radiation exposure has also been associated with an increased incidence of various soft tissue sarcomas. Intracranial leiomyosarcoma was reported 23 years after radiation treatment for a pituitary adenoma [7].

Our review of primary intracranial myomatous tumors found that only one out of 29 reported cases demonstrated smooth muscle differentiation. The other cases were pure mesenchymal or mixed neural and mesenchymal tumors showing skeletal muscle differentiation. Tumors with rhabdomyomatous elements were more common than tumors containing leiomyosarcomatous characteristics [8].

In another study of 3829 patients with soft tissue sarcoma, 21 patients presented with and 19 patients subsequently developed brain metastases. In this study the most frequent tumor type with metastatic brain involvement was leiomyosarcoma [9]. Leiomyosarcomas, however, tend to exhibit hematogenous spread to lung prior to the appearance of brain metastases and, the metastasis usually involves brain parenchyma [9, 10].

The diagnosis of leiomyosarcoma is confirmed by ultrastructural features of smooth muscle cells and immunohistochemistry. The tumor cells are elongated with tapering cytoplasmic processes with elongated, convoluted nuclei, pinocytic vesicles, and basement membrane material around the cytoplasmic membrane. The differential diagnoses, which include malignant astrocytoma, malignant fibrous histocytoma, and meningioma, were excluded by immunohistochemical testing. Our patient's tumor was negative for S-100 protein and epithelial membrane antigen and positive for smooth muscle actin and cytokeratin staining.

Although the pathologic and radiographic examination indicated a primary intracranial leiomyosarcoma in our patient, another primary site could not be excluded with complete certainty. Within the limits of CT scan and other imaging modalities, the evaluation and the clinical course of our patient were consistent with a primary intracranial location.

The prognosis for primary intracranial leiomyosarcoma is poor with the longest reported survival being 32 months [9, 11]. Patient survival is probably limited by the difficulty in obtaining adequate surgical margins and an adequate radiation therapy dose to the intracranial location. Intracranial and meningeal tumor spread may also limit the benefits of systemic adjuvant chemotherapy. Despite these limitations, treatment should probably include aggressive application of multimodality therapy.

A primary intracranial tumor of mesodermal origin is rare and the majority of these tumors are rhabdomyosarcoma. Leiomyosarcomas may mimic meningiomas on preoperative MRI and, although extremely rare, must be included in the differential diagnosis of dural-based lesions

## References

[1] Weitz J, Antonescu CR, Brennan MF (2003). Localized extremity soft tissue sarcoma: improved knowledge with unchanged survival over time. *Journal of Clinical Oncology*.

[2] Paulus W, Slowik F, Jellinger K (1991). Primary intracranial sarcomas: histopathological features of 19 cases. *Histopathology*.

[3] Lee TT, Page LK (1997). Primary cerebral leiomyosarcoma. *Clinical Neurology and Neurosurgery*.

[4] Feigin I, Allen L, Lipkon L, Gross SW (1957). The endothelial hyperplasia of the cerebral blood vessels with brain tumors, and its sarcomatous transformation. *Cancer*.

[5] Brown HG, Burger PC, Olivi A, Sills AK, Barditch-Crovo PA, Lee RR (1999). Intracranial leiomyosarcoma in a patient with AIDS. *Neuroradiology*.

[6] Bejjani GK, Stopak B, Schwartz A, Santi R (1999). Primary dural leiomyosarcoma in a patient infected with human immunodeficiency virus: case report. *Neurosurgery*.

[7] Niwa J, Hashi K, Minase T (1996). Radiation induced intracranial leiomyosarcoma: its histopathological features. *Acta Neurochirurgica*.

[8] Pasquier B, Couderc P, Pasquier D (1977). Les tumeurs ‘musculaires’ ou a composante myosarcomateuse primitives du systeme nerveux central. *Semaine des Hopitaux de Paris*.

[9] Espat NJ, Bilsky M, Lewis JJ, Leung D, Brennan MF (2002). Soft tissue sarcoma brain metastases: prevalence in a cohort of 3829 patients. *Cancer*.

[10] Haykal HA, Wang AM, Zamani A (1987). Leiomyosarcoma metastatic to the brain: CT features and review. *American Journal of Neuroradiology*.

[11] Louis DN, Richardson EP, Dickersin GR, Petrucci DA, Rosenberg AE, Ojemann RG (1989). Primary intracranial leiomyosarcoma. Case report. *Journal of Neurosurgery*.

